# G-Quadruplexes Formation at the Upstream Region of Replication Origin (OriL) of the Pseudorabies Virus: Implications for Antiviral Targets

**DOI:** 10.3390/v13112219

**Published:** 2021-11-04

**Authors:** Yance Zhu, Wenhao Liu, Chao Zhang

**Affiliations:** College of Veterinary Medicine, Henan Agricultural University, Zhengzhou 450046, China; zychenau@163.com (Y.Z.); lwhzk1@163.com (W.L.)

**Keywords:** pseudorabies virus, G-quadruplex, DNA replication, antiviral activity, PhenDC3

## Abstract

Pseudorabies virus (PRV) is the causative agent of Aujeszky’s disease, which still causes large economic losses for the swine industry. Therefore, it is urgent to find a new strategy to prevent and control PRV infection. Previous studies have proven that guanine (G)-rich DNA or RNA sequences in some other viruses’ genomes have the potential to form G-quadruplex (G4), which serve as promising antivirus targets. In this study, we identified two novel G4-forming sequences, OriL-A and OriL-S, which are located at the upstream origin of replication (OriL) in the PRV genome and conserved across 32 PRV strains. Circular dichroism (CD) spectroscopy and a gel electrophoresis assay showed that the two G-rich sequences can fold into parallel G4 structures in vitro. Moreover, fluorescence resonance energy transfer (FRET) melting and a Taq polymerase stop assay indicated that the G4 ligand PhenDC3 has the capacity to bind and stabilize the G4. Notably, the treatment of PRV-infected cells with G4-stabilizer PhenDC3 significantly inhibited PRV DNA replication in host cells but did not affect PRV’s attachment and entry. These results not only expand our knowledge about the G4 characteristics in the PRV genome but also suggest that G4 may serve as an innovative therapeutic target against PRV.

## 1. Introduction

Pseudorabies virus (PRV), a subfamily of *Alphaherpesvirinae*, not only infects its natural host, the pig, but also infects other animals, including wild boars, rodents, ruminants, and carnivores [1,2], which threatens the swine industry. Owing to the wide use of live attenuated vaccines, PRV has been well controlled in most regions of the world. However, since late 2011, some PRV variants have emerged in many provinces of China, and the traditional vaccine has not been able to provide complete protection from these variants, which has led to large economic losses in the pig industry in China [3,4,5]. Some papers have also reported that PRV can infect humans who work on pig farms; the infected patients typically displayed fever, headache, respiratory failure, seizure, and even vision problems [6,7,8]. In particular, the PRV strain hSD-1/2019 was isolated from a patient with acute encephalitis [9], providing direct evidence that PRV has the potential for cross-species transmission from animals to humans. As PRV still exerts a potential threat to the pig industry and public health, it is urgent to explore new strategies to prevent and control PRV infection.

**Figure 1:**
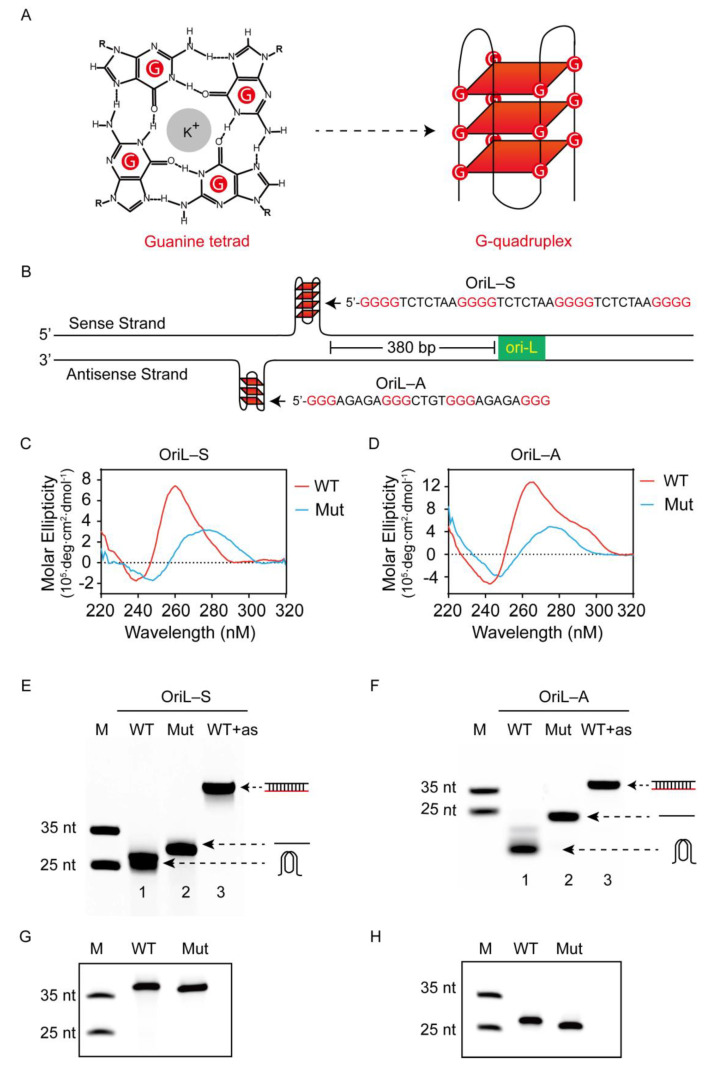
Identification and characterization of G4 formation in vitro. (**A**) A G-tetrad with four guanines connected by eight Hoogsteen hydrogen bonds stabilized by K^+^ and an intramolecular G-quadruplex (G4) composed of three stacking G-tetrads. (**B**) Schematic representation of G-rich sequences at the upstream region of OriL within the PRV genome, OriL-S was located at the sense strand, while OriL-A was located at the antisense strand. (**C**,**D**) Circular dichroism (CD) spectra of wild-type (WT) or mutated-type (Mut) oligonucleotides of OriL-S (5 µM) and OriL-A (5 µM), which were annealed in a buffer with 50 mM LiAsO4 and 100 mM KCl. (**E**,**F**) Wild-type (WT), mutated-type (Mut), and WT complementary with antisense oligonucleotides (as) of OriL-S and OriL-A were subjected to native gel analysis (representative image), the corresponding illustration of G4, liner, and double-stranded DNA was directed by an arrow. (**G**,**H**) WT and Mut oligonucleotides of OriL-S and OriL-A were electrophoresed on denaturing gel with 7 M urea (representative image). All the oligos were labeled with FAM at the 5′-end, and the marker (M) was synthesized with the indicated length but did not form a secondary structure.

The G-rich DNA and RNA are able to fold into a noncanonical secondary structure G-quadruplex, also named G4. G4 is composed of two or more stack G-tetrads; a G-tetrad is connected by four guanine bases via Hoogsteen base pairs and is stabilized by K^+^ (Figure 1A). Increasing evidence indicates that G4s can form in live cells, which have been probed by the single-chain antibody BG4 [10] and confirmed by CHIP-seq [11]. The formed G4s take part in important biological processes, including transcription, DNA replication, translation, and telomere maintenance (as reviewed in [12,13]). Aside from mammalian cells, plants, and prokaryotic cells, an increasing number of studies report that G4 can form in a virus genome (as reviewed in [14,15,16]), such as the human immunodeficiency virus-1 (HIV-1) [17], the Epstein-Barr virus (EBV) [18], the herpes simplex virus (HSV) [19], the hepatitis C virus (HCV) [20], the Ebolavirus disease (EVD) [21], and the Zika virus [22]. In addition, small ligands have been shown to bind and stabilize G4 structures formed in a virus, displaying potential antiviral activity. For example, BRACO19 has the capacity to bind the G4 formed in the HSV genome, thus inhibiting virus replication [19]. Treatment of Ebola with TMPyP4 evidently inhibits L gene expression [21]. Recently, bioinformatics analysis identified G4-forming sequences in the SARS-CoV-2 genome [23,24]. In addition, treatment with PDP reduced the SARS-CoV-2 N expression in vitro and in vivo by targeting the RNA G4 structure in SARS-CoV-2 [25].

Similar to that of HSV, the genome of PRV is characterized by double-stranded DNA, whose G/C content is approximately 70%. We reported a conserved G-rich sequence located in the EP0 promoter able to form G4, which negatively regulated EP0 expression in the presence of the G4 stabilizer PDS [26]. In addition, G4 stacked by two tetrads were identified in the 3′-UTR of IE180 and exhibited a positive regulatory role in IE180 expression [27]. Given the rich G/C content in the PRV genome, we speculated that more G4s might form in the PRV genome and exert biological influence on the PRV’s life cycle.

In this work, we identified two conserved G4-forming sequences at the upstream region of OriL in the PRV genome, which were located in the sense and antisense strands. In addition, multiple biophysical and biochemistry experiments demonstrated that the two G-rich sequences could fold into a parallel G4 structure and be stabilized by PhenDC3 (a G4-targeting compound). Notably, the addition of PhenDC3 into PRV-infected cells inhibited DNA replication in cells. These results indicate that the G4 upstream of OriL may serve as new therapeutic targets against PRV.

## 2. Materials and Methods

### 2.1. Materials and Oligonucleotides

All oligonucleotides and primers used in this work were from Sangon Biotech (Shanghai, China) and are listed in Table 1. TRIzol Reagent (D9108B) and SYBR Premix Ex Taq (RR420A) were obtained from Takara (Dalian, China), the Cell Counting Kit-8 (CCK-8, ZP328) from Zoman Bio (Beijing, China), and the mouse monoclonal anti-actin (A1978) was obtained from Sigma-Aldrich (Beijing, China). Primary antibody Lamin B1 was obtained from Proteintech (Rosemont, IL, USA), Alexa Fluor^®^ 555 conjugated goat anti-rabbit IgG (H+L) was obtained from Thermo Fisher Scientific (Shanghai, China). Anti-glycoprotein E (gE) was a gift from Professor Ke-Gong Tian (Henan Agricultural University, China). PhenDC3 (C103887) was from ChemeGen (Los Angeles, CA, USA). TIANamp Virus DNA/RNA Kit (DP315) was from TIANGEN (Beijing, China).

### 2.2. Cells and Viruses

Pig kidney (PK-15) cells (CCL-33, ATCC) and African green monkey kidney (Vero) cells (CL-81, ATCC) were grown in monolayers at 37 °C under 5% CO_2_. All the cells were cultured in DMEM (10566–016, GIBCO, Shanghai, China), supplemented with 10% fetal bovine serum (10099141C, GIBCO, Shanghai, China), 100 units/mL penicillin, and 100 μg/mL streptomycin sulfate (B540732, Sangon, Shanghai, China).

Han-Zhong Wang (Wuhan Institute of Virology, Chinese Academy of Sciences) kindly donated the recombinant PRV strain of PRV-GFP, derived from the PRV Hubei strain with the TK gene replaced by a GFP expression cassette from pEGFP-N1 plasmid. The PRV HN1201 was a gift from Professor Ke-Gong Tian (Henan Agricultural University, China).

### 2.3. Circular Dichroism (CD) Spectroscopy

This method was conducted as previously described [28]. In brief, wild-type and mutated type single-stranded DNAs (Table 1) were diluted to a final concentration of 1 μM in 50 mM lithium cacodylate buffer (pH 7.4) containing 100 mM KCl. The samples were heated at 95 °C for 5 min and then slowly cooled to room temperature at a rate of 0.01 °C/s. Next, CD spectra were recorded on a Chirascan-plus Circular Dichroism Spectrophotometer (Applied Photophysics, Leatherhead, UK) from 220 to 320 nm at 25 °C with 0.5 mm path length and 1-nm bandwidth. The spectra for all samples were baseline-corrected with buffer and the ellipticities were converted to mean residue ellipticity (θ) = deg × cm^2^ × dmol^−1^ (molellip).

### 2.4. Gel Electrophoresis

For the native polyacrylamide gel electrophoresis (PAGE) assay, wild type and mutated type oligonucleotides (Table 1) were heated at 95 °C for 5 min and then slowly cooled to room temperature at a rate of 0.01 °C/s. Next, the samples were loaded on 20% native polyacrylamide gels containing 100 mM KCl and electrophoresed at 4 °C and a rate of 8 V/cm; the gel was then scanned using an Amersham Imager 600 (GE Healthcare, Boston, MA, USA).

For the denaturing polyacrylamide gel electrophoresis (PAGE) assay, wild type and mutated type 5′-FAM-labeled oligonucleotides (Table 1) were heated at 95 °C for 5 min and then quickly cooled on ice. The samples were separated on a denaturing polyacrylamide gel containing 7 M urea and finally scanned as above.

### 2.5. Taqpolymerase Stop Assay

This method was performed as previously described [29] with little modification. A 1 μM template (WT and Mut ssDNA) and 1.2 μM 5′-Fam-labeled primer (Table 1) were heated at 95 °C for 5 min in a buffer containing 150 mM KCl and 50 mM Tris-HCl and then cooled to 25 °C at a rate of 0.01 °C/s. Then, the indicated concentration of PhenDC3 was added. Primer extension was performed with 2.5 U of Taq DNA polymerase (Takara, Dalian, China) at 60 °C for 30 min. Reactions were stopped by ethanol precipitation, then the extension products were resolved on a denaturing 20% polyacrylamide gel containing 7 M urea. The gel was scanned with Amersham Imager 600 (GE Healthcare, Boston, MA, USA).

### 2.6. Fluorescence Resonance Energy Transfer (FRET) Melting Assay

For the FRET assay, the single-stranded DNA was labeled with FAM (as a donor) at the 5′ end and TAMAR (as a quencher) at 3′ end, respectively. Then, the oligonucleotides were heated at 95 °C for 5 min in buffer (10 mM LiAsO4, 100 mM KCl) and then slowly cooled to 25 °C at a rate of 0.01 °C/s. Then, the indicated concentration of PhenDC3 was added to the sample and incubated at 25 °C for 30 min. After equilibration at 25 °C for 5 min, the melting curves were analyzed on a QuantStudio^TM^6Flex Real-Time PCR System (Life Technologies, Shanghai, China) with the temperature increasing to 99 °C at a rate of 0.01 °C/s.

### 2.7. Cell Viability Assays

PK-15 cells were seeded into 96-well plates with 1 × 10^4^ cells/well. When the cells grew to approximately 70%, the medium was replaced by DMEM/10% FBS supplemented with increasing concentrations of PhenDC3 and incubated for 48 h. CCK-8 (10 μL) was then added to each well, and the cells were incubated for 3 h at 37 °C. The absorbance at 450 nm was detected with a microplate reader (VARIOSKAN FLASH, ThermoFisher Scientific, Shanghai, China).

### 2.8. Viral Titration

Vero cells were seeded at a density of 2 × 10^5^ cells per well in 12-well tissue cultureplates, pretreated with different concentrations of PhenDC3 (1 µM–50 µM) for 1 h, and then infected with PRV HN1201 at a multiplicity of infection (MOI) of 1 plaque-forming unit (PFU)/cell. After 1 h of infection at 4 °C, the cells were washed and maintained in culture supplemented with PhenDC3 (1 µM–50 µM), or DMSO as a nontreated control. At 24 h post-infection (hpi), supernatants were collected for the titration assay.

Viral titration was assessed using the 50% tissue culture infective dose (TCID50) assay. Vero cells were seeded in 96-well plates at a density of 1 × 10^4^ cells per well overnight. The next day, the cells were inoculated with serially ten-fold diluted PRV HN1201 at 37 °C for 1 h. Excess viral inoculums were removed by washing with phosphate-buffered saline (PBS) three times. Then, 200 μL of maintenance medium (1% FBS/DMEM) was added to each well and cultured for another 3–5 days. Cells showing the expected cytopathic effects were counted daily, and the TCID_50_ value was calculated with the Reed-Muench method.

### 2.9. Western Blot Assay

PK-15 cells were cultured in a 60 mm dish and then infected with PRV HN1201 at an MOI of 1. After 1 h of infection at 4 °C, the cells were washed and maintained in a culture medium supplemented with PhenDC3 (1 µM–50 µM) for 24 h. The cells were washed with ice-cold PBS and lysed in RIPA lysis buffer containing 1 mM phenylmethanesulfonyl fluoride (PMSF) for 10 min. After centrifugation at 13,000× *g* at 4 °C for 10 min, the protein supernatant was obtained and quantified using a bicinchoninic acid (BCA) protein assay kit (Sangon Biotech, Shanghai, China). Twenty micrograms of protein were separated in 10% SDS-PAGE gels and then electrotransferred to polyvinylidene fluoride (PVDF) membranes (IPVH00010; Millipore, Shanghai, China). This was followed by blocking in PBS containing 5% nonfat milk and 0.1% Tween 20 for 1 h at 25 °C. Then, the corresponding primary antibodies were incubated at 4 °C for 12 h, followed by incubating secondary antibodies at 25 °C for 1 h. Then, protein blot signals were amplified by using SuperSignal West Femto (34094, Thermo Fisher Scientific, Shanghai, China) and imaged using a chemiluminescence imaging system (GE Healthcare, Boston, MA, USA).

### 2.10. Fluorescent Microscopy

PK-15 cells were seeded into 12-well plates at 2 × 10^5^ cells/well, pretreated with different concentrations of PhenDC3 (1 µM–50 µM), and then infected with PRV-GFP (MOI = 1) for 1 h at 4 °C. The cells were incubated in a maintenance medium (1% FBS/DMEM) supplemented with PhenDC3 (1 µM–50 µM) for 24 h. After washing with phosphate-buffered saline (PBS), the GFP fluorescence was detected with fluorescent microscopy (IX73, Olympus Corporation, Tokyo, Japan).

### 2.11. Flow Cytometry

PK-15 cells were infected with PRV-GFP and where specified, incubated with different concentrations of PhenDC3. The cells were digested with trypsin-EDTA (25200072, GIBCO, Shanghai, China), collected by centrifugation, and suspended in phosphate-buffered saline (PBS). The percentage of GFP-positive cells was measured by flow cytometry on the CytoFLEX instrument (Beckman Coulter, Brea, CA, USA). All data were analyzed with CytExpert software.

### 2.12. Time of Addition Assay (TOA)

The time-of-addition (TOA) assay determineshow long the addition of a compound can be postponed before it loses its antiviral activity. PK-15 cells were seeded into 12-well plates with 2 × 10^5^ cells/well. The cells were infected with PRV-GFP (MOI = 1) for 1 h at 4 °C. After being washed with PBS, a maintenance medium (1% FBS/DMEM) supplemented with PhenDC3 (50 μM) was added every two hours (from 0 to 12 hpi), and cultured for 24 hat 37 °C. The GFP-positive cells were quantified by flow cytometry on a CytoFLEX instrument.

### 2.13. Quantitative Polymerase Chain Reaction (q-PCR)

PK-15 cells were seeded into 12-well plates at a density of 2 × 10^5^ cells/well. The cells were infected with PRV HN1201 (MOI = 1) for 1 h at 4 °C, and then 50 µM PhenDC3 were added. At different time points, cells were collected, and intracellular DNA was extracted using the TIANamp Virus DNA/RNA Kit according to the manufacturer’s instructions. The isolated intracellular DNA was analyzed by real-time PCR using SYBR Premix Ex Taq (Takara, Dalian, China). For quantification of PRV genome copy numbers, the PCR product of PRV gH (187 bp) was cloned into the pGEM-T vector to construct a plasmid pGEM-gH, which was used to prepare a standard curve from serial 10-fold dilutions of pGEM-gH.

### 2.14. Statistical Analysis

Data were obtained from at least three independent experiments for quantitative analyses and are expressed as means ± standard errors of the means. All the statistical analyses were performed with one-way analysis of variance (ANOVA) using prism8 (GraphPad Software, San Diego, CA, USA). Significant differences relative to the corresponding controls were accepted at * *p* < 0.05, ** *p* < 0.01, and *** *p* < 0.001.

## 3. Results

### 3.1. Two Conserved G-Rich Sequences at the Upstream Region of OriL Formed a Parallel G4 Structure In Vitro

The PRV genome is characteristic of G/C-rich content, which led us to search for G4-forming sequences. Interestingly, we found two G-rich sequences located at 380 bp upstream from the origin of replication (OriL) in the viral UL region. One G-core sequence was located in the sense strand of the PRV genome, while another was located in the antisense strand; thus, we named them OriL-S and OriL-A, respectively. In addition, multiple sequence alignment using MEGA6 software [30] revealed that the G-rich sequences of OriL-S and OriL-A were conserved across 32 PRV strains (Supplementary Figure S1). Furthermore, analyzing the G-core sequences of OriL-S and OriL-A with a web-based program QGRS Mapper (http://bioinformatics.ramapo.edu/QGRS/index.php, accessed on 28 October 2019), we found the two sequences theoretically folded into the G4 structure. Specifically, OriL-S’s G4 consisted of four G-tetrads, while the G4 of OriL-A was comprised of three G-tetrads (Figure 1B).

To experimentally test whether G4 formed in both OriL-S and OriL-A, we used circular dichroism (CD) spectroscopy. CD is a universal method to analyze G4 formation and G4 topology. In this experiment, wild-type oligonucleotides (OriL-S-WT and OriL-A-WT) were annealed slowly in 100 mM K^+^ buffer that stabilized the G4 folding. As shown in Figure 1C,D, both wild-type oligonucleotides, OriL-S-WT and OriL-A-WT, displayed a negative peak at ~240 nm and a positive peak of ~260 nm, respectively, which is a typical parallel G4 conformation. In contrast, the mutated type oligonucleotides, OriL-S-Mut and OriL-A-Mut, in which some guanine bases were substituted with adenine bases to disrupt G4 folding, did not show a characteristic G4 structure spectrum.

**Figure 2:**
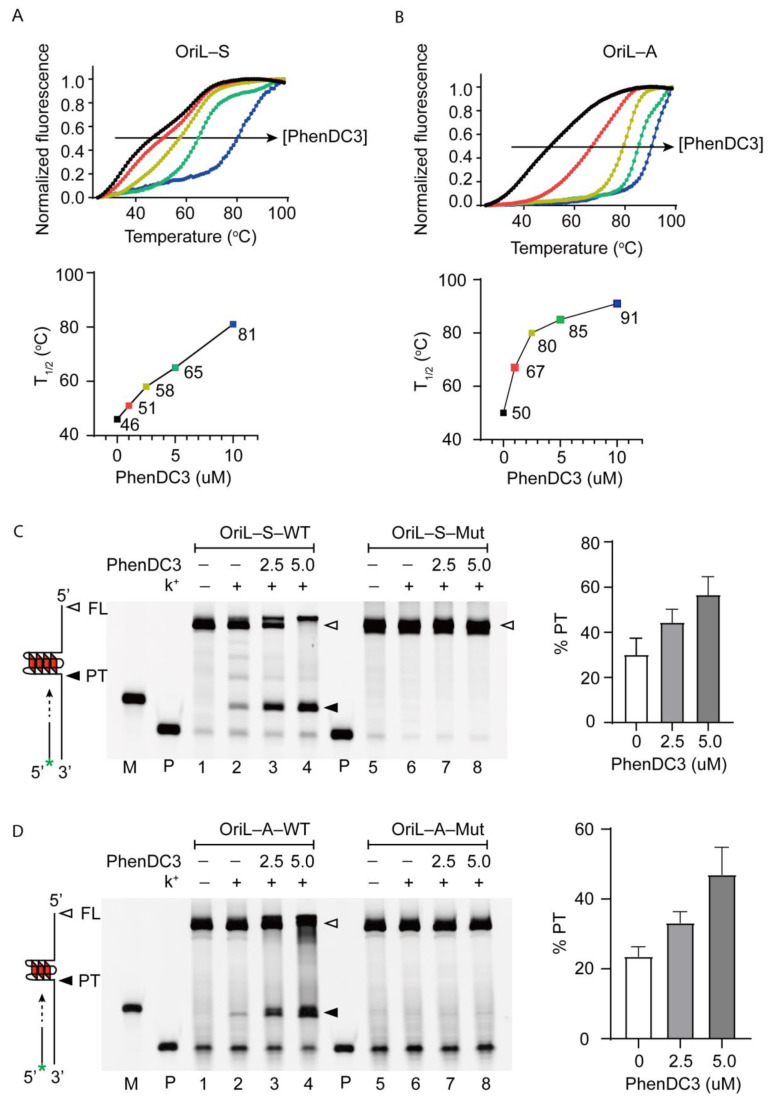
PhenDC3 binds and stabilizes G4 formed in OriL-S and OriL-A. (**A**,**B**) Fluorescence melting curve of OriL-S and OriL-A in the presence of different concentrations of PhenDC3 (horizontal arrow); the T1/2 indicates the temperature required for reaching the mid-value between the minimum and maximum fluorescence in FRET melting, and the T1/2 was plotted against concentrations of PhenDC3 (bottom). (**C**,**D**) Synthesized single-stranded template DNA were annealed with FAM-labeled primers and were extended by Taq polymerase in the absence or in the presence of PhenDC3. Extended products were subjected to denaturing gel analysis (representative image). Premature termination (PT) stopped by G4 and full-length products (FL) were denoted by a filled and open triangle, respectively. P indicates primer used in Taq polymerase stop assay, marker (M) was made by the same extension–reaction with a template without the G-core and its 3′ flanking sequence. The ratio of premature termination (PT) over full-length (FL) products in three independent experiments is demonstrated on the right of the panel.

Next, native polyacrylamide gel electrophoresis (PAGE) was conducted to further confirm G4 folding. A fluorescence FAM was labeled at the 5′ end of the oligonucleotides (oligo) for imaging. After being annealed in 100 mM K^+^, the oligos were subjected to native gel analysis. Because the G4 structure is more compact than linear oligo, oligonucleotides with a G4 structure will migrate faster than their linear counterparts [31,32,33]. As shown in Figure 1E,F, both OriL-S-WT and OriL-A-WT moved faster than the corresponding negative control without G4 (lane one versus two). In contrast, when the two oligonucleotides were annealed with the complementary antisense strand to form double-stranded DNA and to disrupt G4 folding, they migrated more slowly than both the G4-containing DNA and the corresponding linear oligos (lane three). In contrast, when the oligonucleotides were subjected to denaturing gel with 7 M urea, both wild-type and mutated sequences exhibited a similar electrophoresis rate (Figure 2G,H), indicating that they had a similar molecular weight. These results suggest that the different migration rate between WT and Mut sequences in the native gel is related to oligos’ G4 structure but not to oligos’ molecular weight.

Taken together, the above CD and PAGE results reveal that both OriL-S and OriL-A have the capacity to fold into G4 structures.

### 3.2. G4-Stabilizer PhenDC3 Binds and StabilizesG4 Structure of OriL-S and OriL-A

**Figure 3:**
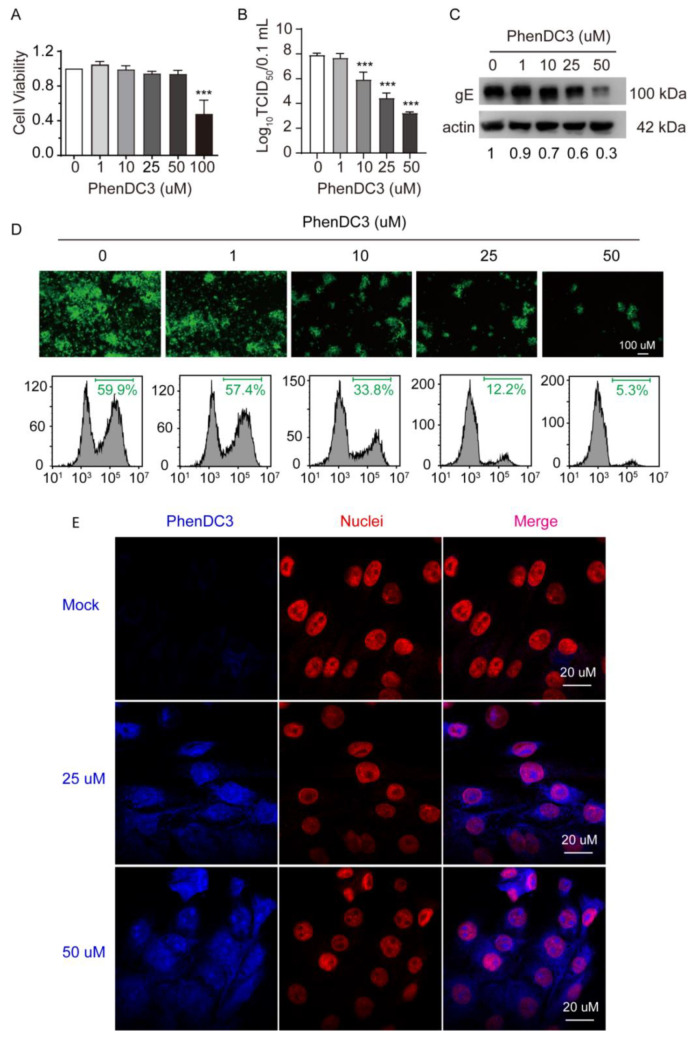
Effect of the G4-stabilizer PhenDC3 on PRV proliferation in PK-15 cells. (**A**) Treating PK-15 cells with increasing concentrations of PhenDC3 for 48 h, the cell viability was assessed by a CCK-8 assay. (**B**) PK-15 cells were pretreated with different concentrations of PhenDC3 (1 µM–50 µM) and then infected with PRV HN1201 at an MOI of 1; after incubating with PhenDC3 for 24 h, the viral titer was determined by TCID_50_ assay. (**C**) PK-15 cells were treated as in (**B**); the expression level of gE was determined by western blot analysis (representative image). (**D**) PK-15 cells were pretreated with different concentrations of PhenDC3 (1 µM–50 µM), and then infected with PRV-GFP (MOI = 0.1); after incubating with different concentrations of PhenDC3 for 24 h, the GFP-positive cells were detected with fluorescence microscopy (top) and quantitatively analyzed by flow cytometry (bottom). (**E**) PK-15 cells were untreated or treated with 25 µM and 50 µM PhenDC3 for 1 h, then the cell nuclei were probed with primary antibody Lamin B1 together with Alexa Fluor^®^ 555 conjugated goat anti-rabbit IgG (H + L). The cells were imaged with a Zeiss LSM 800 Confocal microscopy, λexc = 405 nm was used for imaging PhenDC3, and λexc = 561 nm was used for nuclei visualization. All data are shown as mean ± SD based on three independent experiments. *** *p* < 0.001 determined by one-way ANOVA.

G4-stabilizer PhenDC3 is a well-studied G4 interacting ligand. Therefore, we assessed PhenDC3′s interaction with OriL-S-G4 and OriL-A-G4 by using a FRET melting assay. In this experiment, regarding oligonucleotides labeled with FAM (as a donor) and TAMRA (as a quencher) at the 5′ end and 3′ end, respectively, when the oligos folded into G4 through denaturation/renaturation, the donor came near the quencher and was quenched by it. However, upon G4 unfolding by heating, FAM departed from TAMRA, emitting detectable FAM fluorescence. The middle fluorescence intensity was referred to as T_1/2_, which represented G4′s melting temperature. As shown in Figure 3A,B, in the absence of PhenDC3, the T_1/2_ of OriL-S-WT and OriL-A-WT was 46 °C and 50 °C, respectively. However, when increasing concentrations of PhenDC3 were added, the T1/2 of OriL-S-WT and OriL-A-WT increased substantially to 81 °C and 91 °C, respectively. These results indicate that PhenDC3 is capable of binding and stabilizing the G4 structure formed in OriL-S and OriL-A.

Next, we used the Taq polymerase stop assay to further investigate the interaction of PhenDC3 with the G4 structure. In this experiment, G4 containing single-stranded DNA OriL-S-WT and OriL-A-WT were annealed with FAM-labeled primer in the buffer with or without K^+^ and extended by Taq polymerase. As shown in Figure 2C,D, in the presence of K^+^ that stabilized G4, an obvious stop band was observed compared to the reaction without K^+^ (Figure 2C,D, lane 2 versus lane 1); this might be attributed to the fact that G4 formed in a template hindered Taq polymerase movement, thus producing premature termination (PT) at G4 sites, except for the full-length products (FL). Notably, when PhenDC3 was added, the intensity of the stop band increased dramatically; the ratio of PT increased from 30% to 57% for OriL-S and from 24% to 47% for OriL-A. In contrast, as a negative control, both OriL-S-Mut and OriL-A-Mut did not produce PT whether K^+^ and PhenDC3 were added or not (Figure 2C,D, lane seven to lane eight). These results indicate that G4s are able to hamper polymerase extension along the DNA template.

### 3.3. The Compound PhenDC3 Inhibits PRV Proliferation in PK-15 Cells

Given that OriL-S and OriL-A in the PRV genome have the capacity to form G4 and can be stabilized by PhenDC3, we speculated that PhenDC3 may affect PRV proliferation in cells. First, we tested the cell toxicity of PhenDC3 against PK-15 cells. The CCK-8 assay revealed that PhenDC3 less than 50 µM did not impair cell viability. However, at a high dose, 100 µM PhenDC3 reduced cell viability to ~50% compared to the DMSO vehicle control (Figure 3A). Therefore, PhenDC3 (≤50 µM) was used for the following cellular-based experiments.

To investigate whether PhenDC3 hampers PRV proliferation in PK-15 cells, we treated cells with the indicated concentrations of PhenDC3 and then infected the cells with PRV HN1201 (MOI = 1). After washing away the unabsorbed virus, different concentrations of PhenDC3 were added and incubated at 37 °C for 24 h. The viral titer assay showed that the addition of PhenDC3 substantially reduced viral titer; when 50 µM PhenDC3 was used, the viral titer reduced by approximately 5 TCID_50_ compared to the DMSO control (Figure 3B), suggesting PhenDC3 is able to suppress progeny virion production in host cells. In addition, western blot analysis demonstrated that treatment with PhenDC3 obviously hindered PRV’s gE expression level in cells (Figure 3C), which was consistent with the results obtained from the viral titer assay. To further confirm the impact of PhenDC3 on PRV’s reproduction in cells, PK-15 cells were infected with GFP-tagged PRV (MO1 = 0.1) and then treated with different concentrations of PhenDC3 at 37 °C for 24 h. Obviously, PhenDC3 substantially reduced GFP fluorescence compared with the sample without ligand, as shown by fluorescent microscopy and flow cytometry assay (Figure 3D). Furthermore, using cellular-based fluorescence experiments, we observed a clear fluorescence signal in the PhenDC3-treated PK-15 cells, mainly in the cell nuclei, when compared with the untreated cells (Figure 3E).

### 3.4. G4-Stabilizer PhenDC3 Impairs PRV DNA Replication 

**Figure 4:**
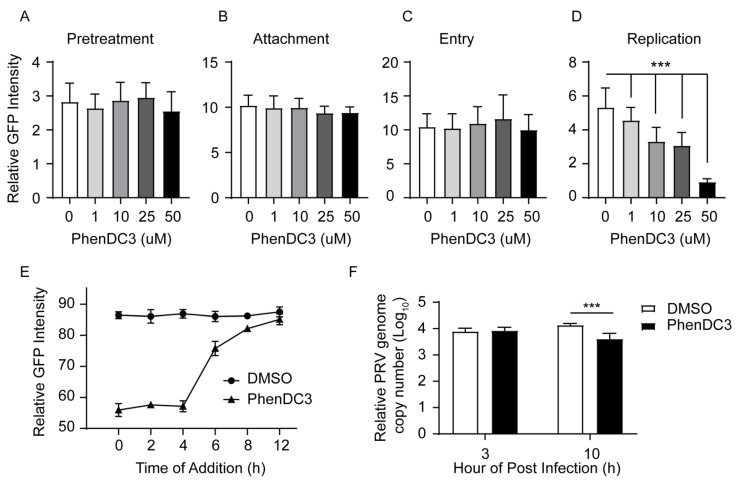
Compound PhenDC3 inhibited PRV DNA replication in PK-15 cells. (**A**) For the pretreatment assay, PK-15 cells were pretreated with increasing concentrations of PhenDC3 (1 μM–50 µM). After removing the PhenDC3, cells were infected with PRV-GFP (MOI = 0.1) for 1 h at 37 °C. Viral inoculums were washed to remove the unabsorbed virus, then the maintenance medium (1% FBS/DMEM) was added and cultured for 24 h at 37 °C. The GFP-positive cells were quantified by flow cytometry. (**B**) For the viral attachment assay, PK-15 cells were treated with different concentrations of PhenDC3 concurrently with PRV-GFP (MOI = 0.1) at 4 °C for 1 h; the excess viral inoculums were removed and replaced with a maintenance medium (1% FBS/DMEM), then shifted to 37 °C and incubated for 24 h. The GFP-positive cells were quantified by flow cytometry. (**C**) For the viral entry assay, PK-15 cells were infected with PRV-GFP (MOI = 0.1) for 1 h at 4 °C. Viral inoculums were washed three times to remove the unabsorbed virus. Different concentrations of PhenDC3 were added and incubated at 37 °C for 1 h to allow viral internalization. The culture was replaced with a maintenance medium (1% FBS/DMEM) and the cells were cultured for 24 h at 37 °C. The GFP-positive cells were quantified as above. (**D**) For viral replication in cells, PK-15 cells were infected with PRV-GFP (MOI = 0.1) for 1 h at 37 °C. After viral internalization, the excess viral inoculums were removed and replaced with a maintenance medium supplemented with different concentrations of phenDC3 (1 μM–50 μM), cells were cultured for 24 h at 37 °C. The GFP-positive cells were quantified as above, with *n* = 3, mean ± s.d., one-way ANOVA, *** *p* < 0.001. (**E**) For the time-of-addition assay of PhenDC3, PK-15 cells were infected with PRV-GFP (MOI = 0.1), 50 µM PhenDC3 was added every 2 h (from 0 to 12 hpi), and then the cells were incubated for 24 h; the GFP fluorescence was determined by flow cytometry. DMSO-treated inoculums were the negative control. (**F**) Quantification of intracellular DNA amounts of PRV HN1201 in PK-15 cells. PK-15 cells were infected with PRV HN1201 (MOI = 1) and treated with 50 µMPhenDC3; at the indicated time point, the intracellular DNA was extracted, and the viral DNA amounts were determined by q-PCR.

Next, we explored in more detail the stage in which PhenDC3 hampered PRV infection in host cells. Among a cascade of infecting stages, PRV first bound to the nectin-1 receptor on the host cells, then entered the cell by fusion of the viral envelope with the host cell membrane [34,35,36].Thus, to investigate whether PhenDC3 affected PRV binding and entry to cells, we carried out a pretreatment assay, a viral attachment assay, and a viral entry assay. Notably, we found that pretreatment of PK-15 cells with different concentrations of PhenDC3 did not affect PRV’s infection ability compared with the DMSO control (Figure 4A). Similarly, the compound PhenDC3 did not affect viral attachment (Figure 4B) and viral entry (Figure 4C) when compared to the DMSO control. In contrast, PhenDC3 substantially impaired PRV replication in PK-15 cells; remarkably, 50 µM PhenDC3 decreased the GFP fluorescence ~5-fold relative to the negative control (Figure 4D). These results indicate that PhenDC3 impairs PRV infecting cells mainly through hindering PRV replication in cells.

To further confirm that PhenDC3 impaired PRV replication in cells, we conducted a time-of-addition (TOA) assay. The TOA assay determines how long the addition of a compound can be postponed before it loses antiviral activity [37], and it has been used to investigate BRACO19’s antiviral action towards HSV-1 [19]. In this experiment, 50 µM PhenDC3 was added to GFP-PRV infected cells every two hours, from 0 to 12 hpi. Then the GFP-PRV amount was measured using flow cytometry. As shown in Figure 4E, notably, from zero to four hpi, compared to the DMSO control, PhenDC3 significantly inhibited the GFP-PRV amount. However, from four to six hpi, the GFP fluorescence increased sharply. From 6 to 12 hpi, it increased close to the DMSO control, indicating that four to six hpi was the last time PhenDC3 exerted inhibitory action, and this time is also the early stage of PRV DNA replication [38]. To confirm these results, we infected PK-15 cells with PRV HN1201 at an MOI of one and added 50 µM PhenDC3 into the viral inoculums. At three hpi and 10 hpi, the PRV genome in PK-15 cells was extracted and quantified by q-PCR, As shown in Figure 4F, at three hpi, the genome copy of PRV was not affected by PhenDC3; however, at 10 hpi, compared with DMSO control, the genome copy numbers of PRV were significantly decreased by PhenDC3, thus supporting the conclusion from the TOA assay.

## 4. Discussion and Conclusions

PRV is the causative agent of Aujeszky’s disease, which causes large economic losses for the swine industry. Of note, since late 2011, some novel PRV variants with enhanced virulence have emerged in pig farms in China, and the traditional live attenuated vaccine could not provide complete protection against the PRV variants. Therefore, it is urgent to find a new strategy to combat PRV. PRV’s genome is characteristic of high GC contents; in this work, we identified two new conserved G-quadruplex forming sequences (OriL-S and OriL-A) at the upstream region of OriL. The two G4-forming sequences conservedacross 32 PRV strains, implying they may have a biological effect on the PRV infection process.

As predicted by the bioinformatics analysis, our biochemistry and biophysical assay demonstrated that the G4 core sequence in OriL-S and OriL-A could form parallel G4 structuresin vitro. Although we do not have direct evidence to verify that G4 actually formed in the PRV genome in live cells due to our limited experimental technology, a previous study demonstrated that PRV’s homolog HSV-1, another member of Alphaherpesvirinae, had G-forming sequences in the inverted repeats [19,39]. In addition, the viral G4s in cells could be visualized with antibody 1H6 and peaked at the time of viral DNA replication [40]. This would help us to further verify PRV G4 forming in cells. Aside from the antibody, some ligands have been developed to selectively bind the G4 structure with high affinity; these ligands normally contain basic aromatic rings, an electron-deficient cationic core, and/or positively charged side chains to facilitate the binding of G4 (as reviewed in [41]). One well-studied G4-stabilizer, PhenDC3, has been widely used to probe G4 formation in vitro and in cellular systems [42,43,44,45]. This ligand binds to G4 through extensive π-stacking on the top G-tetrad [46]. In this work, the FRET melting and Taq polymerase stop assays revealed that PhenDC3 specifically binds and stabilizes G4, and cellular-based fluorescence experiments showed that PhenDC3 is mainly located in the cell nuclei. Moreover, the viral titer assay, fluorescent microscopy, and flow cytometry assay demonstrated that PhenDC3 substantially hampered PRV proliferation in PK-15 cells.

Because PRV infects cells initially through binding receptors on host cells and membrane fusion to entry cells, in this work, the results from the pretreatment assay, viral attachment, and entry experiments to some extent exclude the possibility that PhenDC3 impaired PRV proliferation by disturbing PRV attachment and entry. Furthermore, the time-of-addition (TOA) assay verified that PhenDC3 impaired PRV DNA replication in PK-15 cells. In recent years, increasing evidence has revealed that G4s are implicated in viral transcription, translation, and replication (as reviewed in [14,15,47]). Therefore, these G4 interacting ligands potentially act as antiviral agents. In this work, the G4-stabilizer PhenDC3 was able to hinder PRV DNA replication in cells, suggesting it may serve as an antiviral agent towards PRV.

In the normal DNA replication process, double-helix DNA unwinds to produce single-stranded DNA as a template for primer binding and extending; during this process, the single-stranded DNA have an opportunity to fold into a G4 structure. The G4s have a tendency to obstruct DNA replication through their steric hindrance effect [45,48]. In line with this, a previous study reported that G4s formed in the TR region of Kaposi’s sarcoma-associated herpesvirus (KSHV), blockingthe progression of the replication forks [49]. Similarly, here, G4 formed at the upstream of OriL in the PRV genome may block polymerase from moving, thus impairing PRV DNA replication. To some extent, a Taq polymerase stop assay, which mimics an in vitro DNA replication reaction, could explain the related mechanism, in which the G4 formed in the template stalled Taq polymerase extending, yielding the stop product. Another concern should be considered: in fly, mouse, and human cells, more than 60% of the origins have Origin G-rich Repeated Element (OGRE), which have the potential to form G4s. The formed G4s are involved in DNA replication ignition through binding to some proteins [50,51,52]. In this regard, whether the G4s take part in the replication ignition of OriL needs further investigation.

In conclusion, we identified two conserved G4-forming sequences at the upstream region of OriL; G4-specific compound PhenDC3 inhibited PRV DNA replication in cells. These results not only broaden our knowledge about G4′s distribution in the PRV genome but also imply that G4 may serve as a therapeutic target for PRV.

## Figures and Tables

**Table 1:** Oligonucleotides and primers used in this study.

Assay	Name	Sequence (5′ to 3′) *
CD	OriL-S-WT	**GGGG**TCTCTAA**GGGG**TCTCTAA**GGGG**TCTCTAA**GGGG**
	OriL-A-WT	**GGG**AGAGA**GGG**CTGT**GGG**AGAGA**GGG**
	OriL-S-Mut	AGGATCTCTAAGAAGTCTCTAAGAAATCTCTAGGAAG
	OriL-A-Mut	GAGAGAGAGAGCTGTGAGAGAGAGGA
PAGE	OriL-S-WT	FAM-GGGGTCTCTAAGGGGTCTCTAAGGGGTCTCTAAGGGG
	OriL-A-WT	FAM-GGGAGAGAGGGCTGTGGGAGAGAGGG
	OriL-S-Mut	FAM-AGGATCTCTAAGAAGTCTCTAAGAAATCTCTAGGAAG
	OriL-A-Mut	FAM- GAGAGAGAGAGCTGTGAGAGAGAGGA
	Marker-25nt	FAM-TTTTTTTTTTTTTTTTTTTTTTTTT
	Marker-35nt	FAM-TTTTTTTTTTTTTTTTTTTTTTTTTTTTTTTTTTT
FRET Melting	OriL-S-WT	FAM-GGGGTCTCTAAGGGGTCTCTAAGGGGTCTCTAAGGGG-TAMAR
	OriL-A-WT	FAM-GGGAGAGAGGGCTGTGGGAGAGAGGG-TAMAR
Taq polymerase stop assay	OriL-S-WT	TTTTTGGGGTCTCTAAGGGGTCTCTAAGGGGTCTCTAAGGGGTTTTTCGCA*CTGAGCGAAGATACGGAGCCACGCCA*
	OriL-A-WT	TTTTTGGGAGAGAGGGCTGTGGGAGAGAGGGTTTTTCGCA*CTGAGCGAAGATACGGAGCCACGCCA*
	OriL-S-Mut	TTTTTAGGATCTCTAAGAAGTCTCTAAGAAATCTCTAGGAAGTTTTTCGCA*CTGAGCGAAGATACGGAGCCACGCCA*
	OriL-A-Mut	TTTTTGAGAGAGAGAGCTGTGAGAGAGAGGATTTTTCGCA*CTGAGCGAAGATACGGAGCCACGCCA*
	Primer	FAM-TGGCGTGGCTCCGTATCTTCGCTCAG
qPCR	gH primer F	CTCGCCCTCGTCAGCAA
	gH primer R	GCTGCTCCTCCATGTCCTT

* Gs that take part in G4 folding are shown in bold and the mutated bases are labeled with underline. Bases that annealed to the primer forTaq polymerase stop assay are shown in italics.

## Data Availability

The data presented in this study are available in the article and Supplementary Material.

## Supplementary Materials

The following are available online at https://www.mdpi.com/article/10.3390/v13112219/s1. Figure S1: The conserved G-core sequence of OriL-S and OriL-A.
