# Supplementary material for: G-Quadruplexes Formation at the Upstream Region of Replication Origin (OriL) of the Pseudorabies Virus: Implications for Antiviral Targets
Source: Viruses. 2021 Nov 4;13(11):2219. doi: 10.3390/v13112219 (PMC8623188; doi:10.3390/v13112219)
Supplement: Supplementary file 1 [file viruses-13-02219-s001.zip › viruses-1369848-si.pdf]

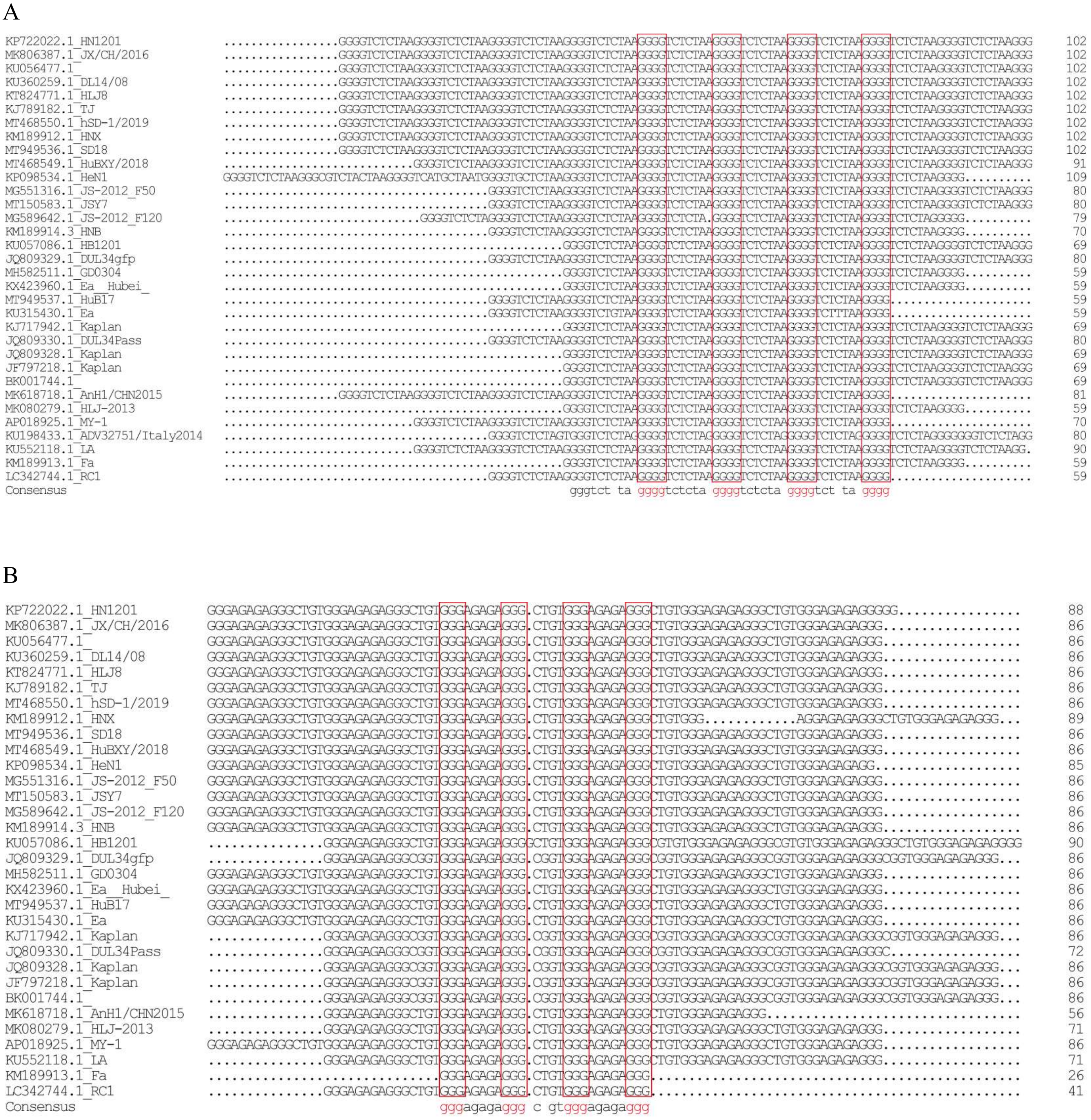

Figure S1. The conserved G-core sequence of OriL-S (A) and OriL-A (B), these sequences were derived from NCBI (www.ncbi.nlm.nih.gov), and MEGA6 software was used for multiple sequences alignment. The consensus G-core sequence was labeled with red.
